# Skin Photoaging and the Biological Mechanism of the Protective Effects of Hesperidin and Derived Molecules

**DOI:** 10.3390/antiox14070788

**Published:** 2025-06-26

**Authors:** Paolo Bellavite, Alice Imbriano

**Affiliations:** 1 Independent Researcher, 37121 Verona, Italy; 2 Pharmacy Program, The Catholic University of the Sacred Heart, 00168 Rome, Italy; alice.imbriano01@icatt.it

**Keywords:** skin photoaging, UV radiation, flavonoids, hesperidin, hesperetin, longevity

## Abstract

The ultraviolet (UV) component of solar radiation is a major risk factor for the development of skin ailments, ranging from erythema in acute cases to premature skin aging and skin cancer in chronic reactions. While skin cells show a remarkable protective capacity against solar radiation, there is a growing interest in the use of natural substances for photoprotection purposes. This article describes the molecular and cellular mechanisms underlying UV radiation-induced skin aging, with a particular focus on the potential beneficial effects of hesperidin and its derivatives: hesperetin, hesperidin glucoside, and hesperidin methylchalcone. A review of the literature from the last 20 years reveals a substantial body of experimental evidence supporting the role of hesperidin in protecting the skin against UV radiation, and its effects on skin cells and tissue, including oxidative stress and aging processes. Moreover, flavonoids have other beneficial effects on skin cell vitality by modulating the immune system, metalloproteinases, and angiogenesis. The key mechanisms for the action of hesperidin and its derivatives involve the activation of the *Nrf-2/ARE* system, the expression of longevity genes *CISD2*, and interference with the *MAP* kinase and *PI3PK/Akt* signal transduction pathways. In murine experimental models, these derivative molecules have a protective role both systemically after dietary intake and through the topical application of dermocosmetic creams.

## 1. Introduction

The skin is the largest organ of the human body and is susceptible to a multitude of external factors and pathogens that have the potential to endanger its wellbeing. Many endogenous and exogenous factors have the potential to impair the highly complex functions of the skin over time. The use of various skin care products, including cosmetics and pharmaceuticals, can serve to minimize or even reverse the effects of these factors.

The skin is continuously exposed to a multitude of external physical, chemical and biological pathogens, which can cause damage such as sunburn, trauma, infection, allergic reactions, and the development of tumors derived from keratinocytes, melanocytes or Merkel cells (MCs). Furthermore, the skin is susceptible to diseases of internal origin, including those related to autoimmunity, metabolic or vascular disorders, metastasis, and the simple “wear and tear of time”.

The sun’s ultraviolet rays are the primary cause of acute skin damage, resulting from the intense and brief exposure of unprotected skin. However, they also act as a catalyst for premature skin aging, leading to a condition known as “photoaging”, which can be defined as a consequence of chronic exposure to solar radiation, particularly ultraviolet (UV) radiation. Moreover, UV irradiation has been shown to induce the neoplastic transformation of skin cells, including keratinocytes and melanocytes.

UV radiation plays its pathogenic role by exerting direct effects on macromolecules, particularly on cellular DNA, and indirect effects through the generation of reactive oxygen species (ROS). These include superoxide anion (O_2_^−^), hydrogen peroxide (H_2_O_2_), hydroxyl radical (˙OH), and lipid hydroperoxide (LO_2_H), as well as nitric oxide (NO) and peroxynitrite (NO_3_^−^). A multitude of cellular and extracellular ROS defense and disposal systems serve to counteract these actions, thereby enabling cells to resist oxidative stress or repair damage.

The effective management of skin conditions is of substantial importance for overall health, quality of life, and psychosocial impact, and thus represents a significant public health concern. In addition to the psychosocial impact and quality of life of affected patients and their families, some chronic skin disorders may also contribute to the development of other systemic diseases. In any case, although skin aging can be considered a normal process, it is a subject of great interest for aesthetic reasons. People attempt to slow down the aging process by preventing pathogenic factors and using active ingredients in skin care formulations.

The pursuit of improved skin health, shaped by collective and individual perceptions, has prompted a shift in focus towards prevention strategies over pathogenic factors and the demand for sustainable skin care products. Skin care products can be classified into distinct categories based on their composition, mode of action, and effects, namely cosmetics, cosmeceuticals, nutricosmetics/nutraceuticals, and pharmaceuticals/therapeutic skin care agents [1].

- The principal objective of cosmetics is to enhance the appearance of the skin; they are incapable of modifying the underlying structure or functionality of the skin. Rather, they provide a transient superficial enhancement, as exemplified by moisturizers;
- The term “cosmeceutical” has been the subject of considerable debate in recent years. Nevertheless, it can be defined as a cross-disciplinary entity, situated at the nexus of the cosmetic and pharmaceutical industries, with the dual objective of combining cosmetic and, to a certain extent, therapeutic benefits for the skin. This results in a range of benefits for the skin, including a reduction in the appearance of wrinkles;
- Nutricosmetics and nutraceuticals, which are products designed for oral ingestion, are employed for the purpose of promoting dermal wellbeing. These products contain molecules that are believed to have a beneficial effect on dermal health when ingested.

Given that exposure to UV light prompts the formation of ROS within the cells of the epidermis and dermis, resulting in oxidative stress and photoaging, there is a considerable focus on the identification of natural antioxidants that can be consumed with food or applied topically [2,3,4]. One of the benefits of the Mediterranean diet in protecting against a number of diseases is the high consumption of foods rich in secondary metabolites, including flavonoids, carotenoids, and ascorbic acid. These are produced by plants as a means of protecting their leaves and fruits from UV radiation, toxicants, and viruses. In a recent survey of ingredients of cosmetic products from 2021 to 2024, polyphenols such as resveratrol, chrysin, and hesperidin methyl chalcone (HMC), were found in 13.2% of anti-aging formulations [5].

Hesperidin, a promising flavanone glycoside, and its derivatives can be ingested as part of a regular diet rich in citrus fruits or as dietary supplements. In addition to the well-documented benefits for cardiovascular function [6,7], type II diabetes [8,9], neuroprotection [10,11], and viral diseases [12,13], recent studies in experimental models have demonstrated the multiple benefits of hesperidin for skin functions, including wound healing, UV protection and anti-inflammatory, antimicrobial, and antitumor action. Furthermore, they are extensively employed in dermocosmetics, either as pure substances or in complex formulations.

This article reviews the fundamental mechanisms by which UV damage occurs at various layers of skin tissue and the effects of citrus flavonoids, particularly the flavanone glycoside hesperidin, on skin health. Most of the studies found in the literature used cellular models or experimental animals, even if some research on humans is starting to appear. A pilot study on 30 human subjects reported that a cream comprising a combination of natural substances, including glucosyl hesperidin, resveratrol, niacin amide, and N-stearoyl-D-erythro-dihydrosphingosine, led to an improvement in skin hydration as early as one hour after application. Furthermore, the cream was observed to reduce the appearance of wrinkles and enhance skin elasticity when applied for a period of four weeks [14]. Mandarin peel, containing high amounts of hesperidin, has been traditionally used as a cosmetic ingredient, and experimental evidence shows that it is able to increase the production of hyaluronic acid by skin cells such as fibroblasts and keratinocytes [15]. For this reason, numerous studies are underway to determine the best formulations for inserting hesperidin or its derivatives into cosmetic formulations [16,17,18,19,20].

## 2. Skin Structure Basics

**Figure 1:**
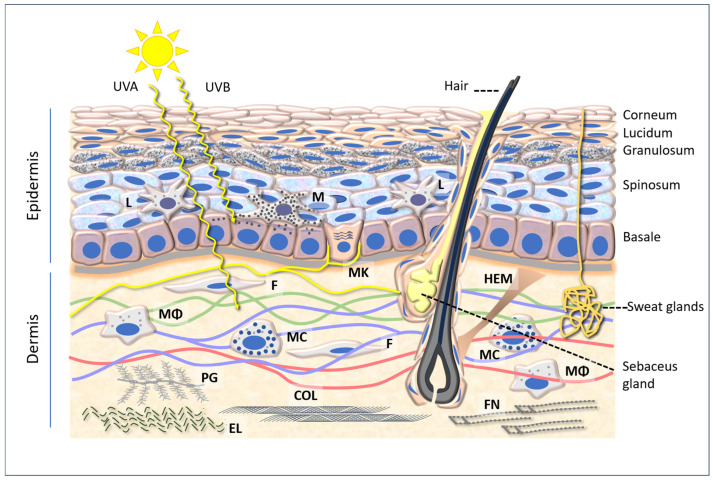
A diagram of the epidermis and dermis, showing the penetration of UVA (315–400 nm) and UVB (280–315 nm) and several cellular and intercellular components referred to in the text. Abbreviations: L: Langerhans cells; M: melanocyte; MK: Merkel cell; F: fibroblast; HEM: hair elevator muscle; Mϕ: macrophage; MC: mast cell; PG: proteoglycan; COL: collagen; EL: elastin; FN: fibronectin.

The skin is the largest organ of the human body, serving as the primary interface with the external environment. It fulfils a number of functions, including mechanical protection, acting as a barrier against physical and chemical agents, thermal and tactile sensitivity, thermoregulation, and immune function. These valuable functions are enabled by the skin’s intricate anatomical structures, which can be broadly classified into three layers: the epidermis, dermis, and hypodermis, which vary in structure and composition (Figure 1).

### 2.1. Epidermis

The epidermis, the outermost layer of the skin, is composed of approximately 90 percent keratinocytes, which are distinguished by their remarkable capacity for keratin synthesis. The group of sulfur-rich fibrous proteins known as keratins is responsible for imparting the tough, water-resistant properties observed in hair, nails, and skin. In addition to intra- and intermolecular hydrogen bonds, keratins contain a substantial quantity of the sulfur-containing amino acid cysteine, which forms disulfide bridges that contribute to the overall strength of the protein. Keratins exhibit an alpha-helical secondary structure. Two keratin proteins unite to form a quaternary structure, a spiral dimer, whereby the helices wrap around themselves. Subsequently, the dimers assemble into protofilaments, which then coalesce to form filaments. The configuration of the twists is analogous to that of the fibers within the strings. The self-assembly of keratins is contingent upon their primary structure.

The epidermis is structured in five layers, which rest on a basement membrane that separates them from the dermis. The stratum corneum, comprising scales of keratinocytes that have undergone apoptosis, plays a pivotal role in the epidermis, providing a protective barrier against the external environment. Keratinocytes are interspersed with other cells, including Langerhans cells, which constitute the initial active barrier against the penetration of microorganisms. These cells are part of the phagocyte system, which includes cells that engulf and destroy bacteria and present antigens to the immune system. Furthermore, melanocytes, which produce melanin, are present and are responsible for the dark coloration of the skin. Melanocytes are derived from neural crest cells and differentiate into melanoblasts, which migrate dorsolaterally towards the ectoderm (the outermost layer of the embryo) where they undergo differentiation into melanocytes. Melanocytes are dendritic-shaped cells that are located in the epidermis, together with keratinocytes. They account for approximately 8% of the cells in the epidermis [21,22].

Melanocytes are characterized by a high concentration of melanosomes, which are melanin-rich organelles that can also be secreted from the cell into the surrounding environment where they can be transferred to neighboring cells [23]. This process results in the formation of a protective cap around the nucleus, which serves to shield it from UV-induced DNA damage. This is of particular importance for basal stem cells, as it prevents both carcinogenesis and apoptosis, and maintains a healthy stem cell pool for the purposes of maintaining skin homeostasis [22].

Melanin is considered to be a double-edged sword. While it serves to protect the epidermal cell nucleus from UV radiation, an excess of melanin can become a source of ROS [24]. It is therefore evident that there is fundamental importance attached to the cellular detoxification systems and the supplementation of antioxidant and UV-protective agents.

Furthermore, a number of MCs are located in the basal layer of the epidermis, situated within the dermal–epidermal junction. It is hypothesized that these cells play a role in sensory discernment and are in contact with afferent sensory endings [25]. They have greater density on the lips, in the palate, on the palms and fingertips, and on the dorsum of the feet [26]. Merkel cell carcinoma (MCC) is an aggressive skin cancer for which UV rays are among the main causative factors [27].

### 2.2. Dermis

The dermis of the skin is composed of two distinct layers of connective tissue. The papillary layer is composed of loose connective tissue, whereas the reticular layer, situated in a position beneath it, is comprised of dense, irregular connective tissue. This region of the dermis contains both blood and lymphatic vessels, as well as nerves. The dermis is populated with fibroblasts, which are responsible for the production of collagen and elastin fibers within the extracellular matrix. The fibroblasts are distributed throughout the collagen and elastin fibers of the connective tissue of the papillary layer. The dermal papillae, situated in close proximity to the blood capillaries, intertwine with the epidermal ridges of the basal layer. The layer is composed of a network of collagen and elastin fibers. Fibroblasts form collagen bundles that extend into the papillary layer and hypodermis, thereby rendering these layers difficult to distinguish. Flexible collagen provides the skin with structural integrity and strength, while elastin confers limited elasticity.

Water is a vital component for maintaining the optimal elasticity and turgidity of the skin; however, it is not readily accessible in the dermis. Instead, it is attached to the polysaccharides of proteoglycans. The accumulation of fluid in the dermis gives rise to the phenomenon of “oedema,” which may be attributable to disturbances in the hydro-electrolyte balance (e.g., diseases of the heart, kidney, or liver) or inflammation.

The extracellular matrix (ECM) of the dermis plays a pivotal role in facilitating cellular attachment and communication with neighboring cells, thereby influencing cell growth, cell movement, and the repair of damaged tissue. The inner dermis is composed of an ECM that is rich in collagen (predominantly types I and III), elastin, fibronectin, and vitronectin. The ECM is responsible for promoting the anatomical stability of the skin. However, it is commonly disrupted in aged skin. For example, solar elastosis is a condition characterized by the accumulation of dysfunctional elastin fibers in response to increased levels of sun exposure, which in turn leads to the formation of fine wrinkles [28].

In addition to fibroblasts, which are responsible for the synthesis of collagen, elastin fibers, and proteoglycans, the dermis of the skin contains a variety of other cell types (Figure 1). Such cells include macrophages, which are responsible for phagocytosis and the killing of microorganisms. Furthermore, macrophages are responsible for the synthesis of fibronectin, a protein that plays a pivotal role in immune functions and wound healing [29,30]. Mast cells, conversely, are typical cells with the capacity to release histamine in response to a range of chemical, physical, and immunological stimuli.

The subcutaneous tissue contains adipose tissue, which serves as an energy reserve, insulates the body, and prevents heat loss. The distribution of adipose tissue differs significantly between genders, lifestyles, and with age. The most effective means of controlling body fat accumulation is through a balanced diet and regular exercise, particularly when body fat levels reach a level that increases the risk of cardiovascular disease.

## 3. Aging of the Skin

**Figure 2:**
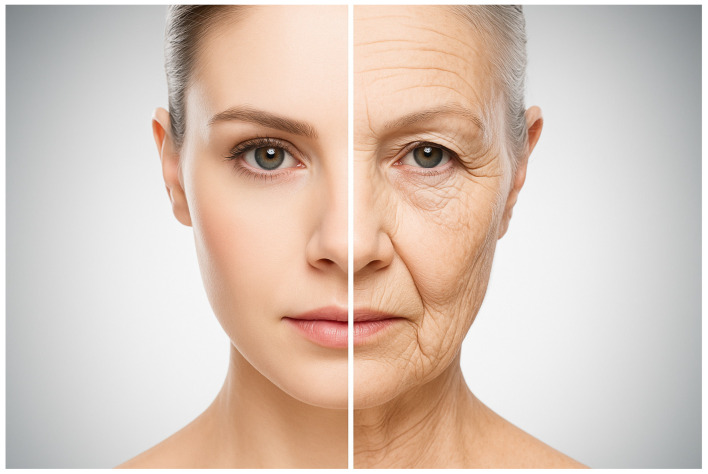
An effective representation of skin aging. An AI image generated by the authors.

The process of aging is a complex physiological phenomenon that results in functional and aesthetic alterations in skin tissue. These changes can be accelerated by a number of factors, including chemical (such as elevated blood glucose levels) and physical (such as sun exposure) influences. The process of aging affects the flexibility of the musculoskeletal system, including tendons and ligaments, as well as the flexibility of vessels. The latter can result in the formation of plaque within the arteries, a condition known as arteriosclerosis. In the skin, the visible effects of aging are manifested in the form of wrinkles and fibrotic hardening of the dermis, as well as alterations in the epidermis and underlying connective tissue. Although these are common experiences and observations, Figure 2 (constructed with AI) illustrates the external signs of skin aging.

The external manifestations of cutaneous aging are well documented and include the formation of wrinkles on the forehead, lateral aspects of the face, and the hands, particularly in regions of the skin that are frequently exposed to solar radiation. The lips may also be affected, presenting with cracking and thinning of the skin. Telangiectasias (spider veins) may develop around the nose and cheeks, while sun freckles (age spots) and precancerous lesions (actinic keratoses) may also occur. The epidermis undergoes a reduction in thickness and an alteration in the organization of collagen fibrils in the dermis [31], accompanied by a decrease in elastin fibrils [32] and the proteoglycan network, which are complex macromolecules with significant functions in the natural properties and appearance of the skin [28].

A comprehensive and compelling account can be found in an article in the New England Journal of Medicine titled “Unilateral dermatoheliosis”, which is available via open access [33]. The photograph depicts a 69-year-old male truck driver who, for several years, while driving, exposed his face to light asymmetrically. The individual in question reported a 25-year history of gradual thickening of the skin and the appearance of more prominent wrinkles on the left side of the face. Histopathological analysis revealed the degradation of elastin in the dermis and the formation of granules within the hair follicles of the vellus. The patient reported driving a delivery truck for 28 years.

**Figure 3:**
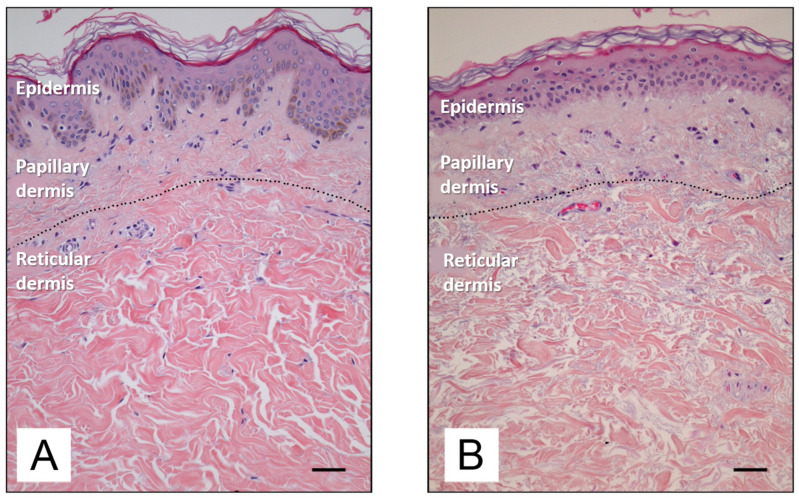
Histological features of young (**A**) and old (**B**) human skin [35]. Available via Creative Commons license BY 4.0. Scale bar: 50 mm.

Figure 3 depicts the difference between young and old skin at the histological level. As the skin ages, there is a reduction in epidermal proliferation, impaired lipid synthesis and barrier function, and an increase in surface pH [34]. In the dermis, there is a reduction in collagen and elastin synthesis, accompanied by an increase in the enzymatic degradation of these proteins by numerous metalloproteinases (MMPs) [1]. MMPs constitute a family of enzymes belonging to the protease group, the catalytic mechanism which requires the presence of metal ions as cofactors. The majority of these enzymes are zinc-dependent proteinases, although a minority utilize cobalt. MMPs play a physiological role in the turnover of normal tissues and the degradation of the extracellular matrix, thereby facilitating the movement of cells such as leukocytes. However, MMPs have also been linked to the processes of ageing and cancer cell metastasis.

It is widely accepted that the overexpression of MMP-1 and MMP-3 is responsible for the formation of wrinkles and the sagging of the skin [36]. MMPs typically facilitate natural matrix remodeling; however, when produced in excess by skin cells or macrophages in the dermis, they contribute to degeneration and accelerate the ageing process. In addition to protein loss, ECM degradation includes oxidative stress [37], which entails both augmented ROS production and diminished enzymatic and non-enzymatic protective mechanisms [38]. The deterioration of tissue structure results in a loss of skin firmness and elasticity, as well as the formation of wrinkles and an enlargement of sebaceous gland pores.

During the process of aging, the dermal ECM undergoes structural alterations, including the degradation of collagen and elastin fibers, as well as hyaluronic acid. As a consequence of the aging process, there is a decline in the population of dermal fibroblasts and their capacity to support epithelial tissue growth [35]. A reduction in the number of fibroblasts also contributes to alterations in the ECM and its subsequent degradation, which results in progressive skin thinning, increased wrinkles, and a loss of elasticity. The activation of pro-inflammatory cytokines, among other factors, can result in the development of dermatological complications, including skin inflammation, wrinkles, a loss of skin elasticity, hyperpigmentation, and dehydration.

The molecular basis of skin aging is highly complex and involves the participation of several genes, including CDGSH iron–sulfur domain-containing protein 2 (*CISD2*), which has been identified as a pro-longevity gene that plays a pivotal role in regulating health span in mammals [39]. In human skin, *CISD2* is predominantly expressed in proliferating keratinocytes of the basal layer of the epidermis. Furthermore, *CISD2* is upregulated in sun-exposed epidermis [40]. *CISD2* plays a pivotal role in regulating lifespan by maintaining mitochondrial function, endoplasmic reticulum integrity, intracellular Ca^2+^ homeostasis, and the redox state [39].

The process of skin aging is the result of a combination of intrinsic and extrinsic factors [40,41]. The process of intrinsic skin aging is associated with a number of factors that naturally change with age. Such factors include a reduction in cell proliferative capacity, a reduction in antioxidant capacity, and disturbances in metabolic homeostasis. Extrinsic skin aging is induced by environmental factors, including air and water pollution, but particularly chronic UV exposure in sun-exposed areas such as the face and neck.

## 4. Solar Radiation Pathology

**Figure 4:**
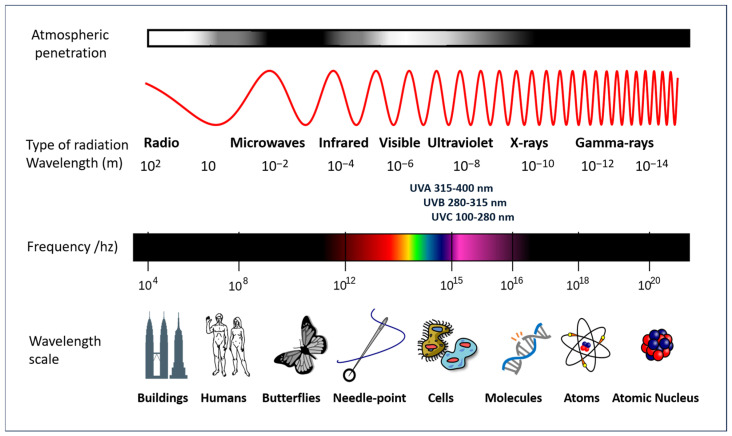
A diagram of the electromagnetic spectrum. Adapted from a NASA image (GNU Free Documentation License).

The Earth’s biosphere is dependent on the sun’s radiant energy, which is capable of originating electromagnetic radiation which encompasses a wide range of frequencies, including both visible and invisible forms. To elaborate further, the solar radiation that traverses the Earth’s atmosphere is composed of the following types of electromagnetic radiation: visible light (approximately 37% of the total electromagnetic radiation reaching the planet’s surface), infrared rays (approximately 60% of the radiation), and UV radiation (approximately 3% of the radiation). Furthermore, shorter wavelength radiation (ionizing) and longer wavelength radiation (microwaves and radio frequency) are present in smaller amounts, due in part to the ozone layer that surrounds the planet. These forms exhibit distinct characteristics and properties, as illustrated in Figure 4.

While the lowest levels of UV radiation reach the Earth’s surface, they are of particular interest due to their role in the tanning effect and their primary responsibility for photoaging and other forms of damage, including those that are severe, caused by inappropriate exposure to sunlight. The wavelength of UV radiation falls within the range of 100–400 nm and is further subdivided into three categories: UVA (315–400 nm), UVB (280–315 nm), and UVC (100–280 nm). The UV component of Earth’s radiation from the midday sun is comprised of approximately 95 percent UVA and 5 percent UVB. The majority of UVB is removed from extraterrestrial radiation by stratospheric ozone. Conversely, UVA is also capable of penetrating glass and is more readily absorbed by the deeper layers of the skin.

In humans, sunlight is beneficial for vision and for maintaining thermal homeostasis through infrared radiation. Moreover, UV light is of particular importance for synthesizing vitamin D, which has hormone-like functions, including the intestinal absorption of calcium and phosphate, and the renal reabsorption of calcium [42]. Furthermore, vitamin D plays a role in maintaining immune system equilibrium [43]. In addition to the aforementioned effects, UV radiation has been demonstrated to possess beneficial properties, including the ability to eradicate pathogenic microorganisms and facilitate the healing process in conditions such as psoriasis.

Nevertheless, excessive exposure to solar radiation can result in acute and chronic detrimental effects on the health of the skin, eyes, and immune system. Exposure to high doses of sunlight in a short period of time, particularly if the skin is unprotected and has minimal melanin, results in acute erythema of varying severity. This is due to the dilation of the microcirculation of the papillary dermis and the subsequent production of inflammatory substances by keratinocytes. In cases of severe sunburn, the formation of serous blisters and ulceration may occur within a few hours of exposure.

As already shown in Figure 1, UVA radiation has a greater capacity to penetrate the dermis than UVB radiation and is thus responsible for the formation of wrinkles and other signs of premature ageing. UVB radiation affects the upper layers of the skin, primarily responsible for changes on the skin surface, such as sunburn and skin darkening associated with sunburn and tanning. Due to its shorter wavelengths, UVB radiation is predominantly absorbed by the epidermis, resulting in direct damage to DNA and cells. In contrast, UVA is less energetic and has a greater penetration depth, reaching both the epidermis and dermis, where it causes oxidative damage to fibroblasts [44].

The administration of high doses of UV radiation (200–500 mJ/cm^2^) over a short period of time has been observed to result in the degranulation and subsequent inflammation of mast cells, accompanied by the accumulation of oedema [45]. Conversely, the same study demonstrated that lower doses (10–100 mJ/cm^2^) do not induce irritation but, conversely, inhibit degranulation induced by the mast cell activating compound 48/80. The results indicate that UVB radiation exerts a dual effect on mast cells, contingent upon the dose, a finding that has been corroborated by other studies [46].

As a consequence of chronic UV exposure, a number of adverse effects may occur, including hyperkeratosis, dyschromia (solar freckles or yellowing), a loss of elasticity, skin thinning, wrinkles, dryness, and other signs of photodamage and skin ageing. In the dermis, a pathological condition has been identified and termed cumulative solar damage [47]. This degenerative condition primarily affects the connective tissue of the dermis and its components, including collagen and elastin, which are damaged by exposure to UV radiation. This condition is particularly prevalent in the elderly and in individuals who, for various reasons (occupational or otherwise), are chronically exposed to solar radiation.

Among the most serious consequences of prolonged sun exposure, particularly when uncontrolled and irresponsible, are skin cancers. While cancers are diseases with multifactorial causes, it is established that UV radiation can increase their risk of occurrence [48]. All these alterations overlap with the characteristics of ageing, which, if on the one hand is so inevitable as to be considered physiological, on the other hand can be accelerated and manifest as pathological processes.

### 4.1. Molecular Mechanisms

**Figure 5:**
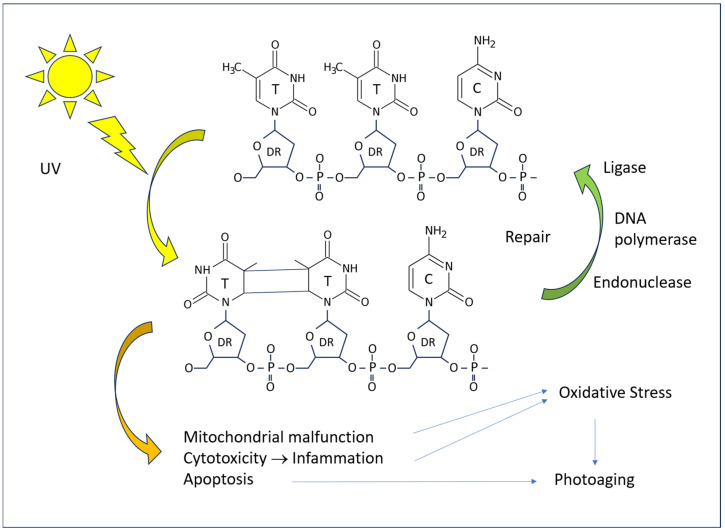
A diagram of the effect of UV radiation on human DNA and consequences on the cell.

The pathogenic effects of excessive UV radiation manifest at the cellular and tissue levels through direct and indirect mechanisms. In the absence of repair, DNA damage results in a reduction in protein production, the initiation of apoptotic processes, and, in the event of survival, the introduction of mutations. Both types of UV rays have an excitatory effect on the DNA of skin cells, with a particular impact on pyrimidine bases at the molecular level (Figure 5). The most notorious form of damage is the formation of pyrimidine dimers (T-T thymine dimers, T-C thymine–cytosine dimers, and C-C cytosine–cytosine dimers) when two of such bases are adjacent in the chain of a single DNA strand. Such damage is a common occurrence in tissues exposed to sunlight. However, the vast majority of such lesions are repaired rapidly, with only a small proportion leading to pathological cell alterations.

The repair of T-T dimers in humans is a process that involves the excision of a portion of the DNA strand, facilitated by the action of an endonuclease. The resulting gaps are then filled with new bases, which are inserted by DNA polymerase I. Finally, DNA ligase seals the insert into the DNA. Defects in the DNA repair mechanisms have a deleterious effect on the skin. The most well-known of these is xeroderma pigmentosum, which is characterized by frequent photosensitization dermatitis and an increased incidence of skin cancer [49]. These patients are primarily affected by UV-induced thymine dimers, but there is growing evidence that the genetic disease also manifests in the defect of protection against other types of biochemical injury, including oxidized DNA bases [37].

### 4.2. Cellular Mechanisms

**Figure 6:**
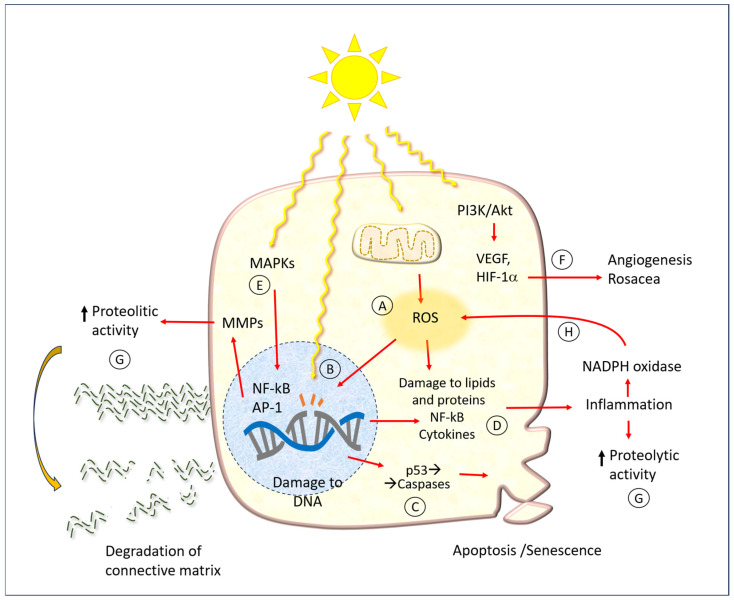
A diagram of cell and tissue damage caused by UV radiation. Abbreviations: MMPs: matrix metalloproteinases; ROA: reactive oxygen species; ECM: extracellular matrix; MAPKs: mitogen-activated protein kinases; PI3K: phosphatidylinositol 3-kinase; Akt: protein Aks, or protein kinase B; VEGF: vascular endothelial growth factor; HIF-1α: hypoxia-inducible factor; NF-kB: nuclear factor kappa-light-chain-enhancer of activated B cells; AP-1: activator protein transcription factor; NADPH: nicotine adenine dinucleotide phosphate. Capital letters indicate the main action points of hesperidin and related molecules, as mentioned in the text. The red arrows represent the cause-effect relationships between the various factors indicated here.

In addition to damage to DNA, UV radiation has a wide array of consequences on various biochemical cell function mechanisms, which are summarized schematically in Figure 6. The same figure shows the points where the protective molecules described later in the text act.

One of the earliest mechanisms of photodamage is the activation of mitogen-activated protein kinase (MAPK), which in turn regulates the expression of the nuclear factor kappa-light-chain-enhancer of activated B cells (NF-κB) and activator protein-1 (AP-1). It has been demonstrated that AP-1 and NF-κB activate MMPs in both the dermis and epidermis [36]. This results in damage to both collagen and elastin within the extracellular matrix of the dermis. Conversely, the MAPK pathway has been linked to the expression of pro-inflammatory cytokines. Such phenomena may manifest acutely following extensive sun exposure of unprotected skin, or they may occur more insidiously, contributing to the development of chronic damage associated with the typical aging process.

In a number of mammalian skin models, the underlying pathogenic mechanism appears to be the formation of free radicals, which can induce ROS and oxidative stress [50]. In physiological conditions, each cell produces a specific amount of ROS through various processes, including enzymatic oxidation-reduction reactions, the oxidative phosphorylation of mitochondria, the metabolism of xenobiotics, and the activity of innate immune defense cells (O_2_^−^-producing NADPH oxidase).

A multitude of enzymatic and non-enzymatic disposal processes serve to maintain the delicate equilibrium of cell functions. In normal conditions, cells do not suffer significant damage, except perhaps slight and progressive molecular alterations that affect the aging process. When ROS levels are elevated, they react with biological molecules, resulting in mutations to nucleic acids, damage to proteins (particularly those containing sulfur-containing amino acids), and alterations to cell membranes. It is hypothesized that the intracellular ROS produced in UV-exposed keratinocytes are primarily derived from the mitochondria and the NADPH oxidase (NOX) enzyme complex [36]. In comparison to individuals with fair skin, those with darker skin types produce a lesser quantity of radicals in UV light, while exhibiting a similar level in visible light. Moreover, UV radiation impairs the activity of ROS disposal mechanisms, such as catalase [51].

Couperose, more commonly known as rosacea, is a chronic benign dermatitis characterized by the presence of dilated capillaries. It is prevalent in individuals with fair complexions, particularly the face of women. The precise pathophysiology of rosacea remains unclear. It is hypothesized that a complex interplay of UV light, neuronal and vascular dysfunction, and immune system alteration, with a genetic component, is involved in the etiology of rosacea [52]. It is thought that UV light plays a role in all aspects of rosacea, including neoangiogenesis, telangiectasia, and fibrosis. Indeed, there is evidence to suggest that UV light may even act as a trigger for rosacea.

The biochemical mechanism of the angiogenic effect of UV radiation also occurs via the phosphoinositide 3-kinase (PI3K)/Akt pathway. In short, PI3K phosphorylates phosphatidyl inositol 4,5 bisphosphate (PIP2), which is converted to phosphatidyl inositol 3,4,5 triphosphate (PIP3). This subsequently results in the activation of free Akt within the cytoplasm. Once activated, Akt phosphorylates a number of cellular protein targets, thereby inducing cell growth. In the context of UV-induced angiogenesis or rosacea, this ultimately leads to the net induction of cell growth.

## 5. Hesperidin and Related Molecules

A multitude of natural substances have been utilized in the field of skin care and protection from UV light. Of particular note are polyphenols, which are organic compounds produced by plant metabolism (and are therefore referred to as “secondary metabolites”). A substantial body of evidence attests to the numerous health benefits associated with polyphenols, which are consequently employed as valuable food elements or incorporated into skin creams [53,54]. The plant kingdom is home to thousands of different molecules, each with its own distinctive phytochemical properties. Such compounds include those that defend against bacterial, viral, and fungal infections, as well as those that regulate insect interactions and mammalian feeding.

Dietary polyphenols represent a diverse group of compounds with the potential to exert beneficial effects in a range of communicable and non-communicable diseases. Epidemiological studies have demonstrated that these substances are associated with increased longevity and a reduced incidence of cardiovascular disease and cancer in the populations that consume them [55,56,57,58,59,60]. There is also a large body of literature, especially in experimental models, which attests that flavonoids such as the flavanone glycoside hesperidin and the flavonol quercetin have beneficial effects on neurodegenerative diseases such as Parkinson’s and Alzheimer’s, which are associated with aging [11].

In the search for natural ingredients with protective effects for the skin, hesperidin appears to be a promising candidate due to its diverse range of potentially beneficial properties, including antioxidant, photoprotective, anti-inflammatory, anticarcinogenic, and antibacterial activities [1,61,62]. Hesperidin is generally regarded as safe for both topical and systemic administration [63,64]. It has been demonstrated that the topical and systemic administration of hesperidin can positively impact a range of dermal functions in both healthy and diseased skin [61]. The topical administration of 2% hesperidin for a period of nine days did not result in any adverse cutaneous reactions in mice [34].

### 5.1. Chemistry of Hesperidin

**Figure 7:**
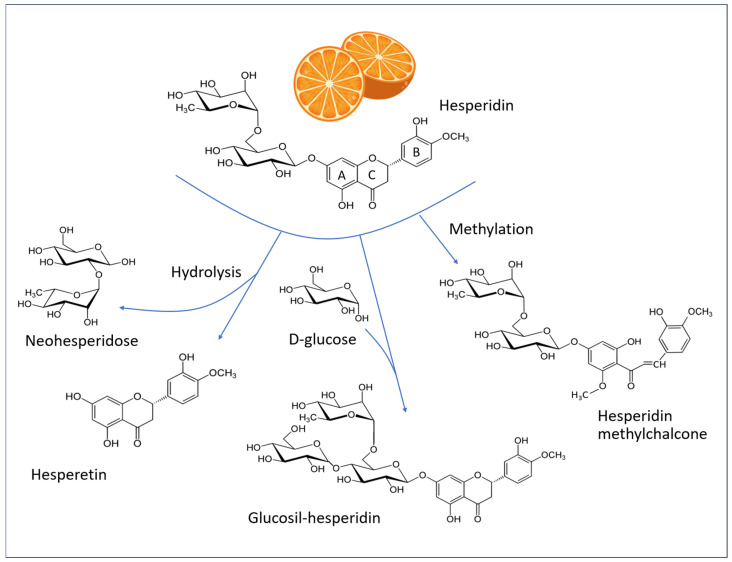
Structural formulas of hesperidin (C_28_H_34_O_15_, MW 610.6 g/mol), hesperetin (C_16_H_14_O_6_, MW 302.27 g/mol), alpha-glucosyl hesperidin (C_34_H_44_O_20_, MW 772.7 g/mol), and hesperidin methylchalcone (C_29_H_36_O_15_, MW 624.6 g/mol). A and B: Phenyl rings, C: heterocyclic ring of the flavone backbone.

Flavonoids represent the most prevalent class of polyphenols in the Mediterranean diet. The term “flavonoid” comes from the Latin flavus, yellow. This is due to the fact that a significant proportion (though not all) of these compounds exhibit a yellow coloration. These plant pigments possess a basic structure comprising a skeleton of 15 carbon atoms (C-15) arranged in three rings: two benzyl rings (A and B) and a heterocyclic ring (C). Figure 7 illustrates the chemical structures of hesperidin and related molecules that are referenced in the text.

Flavonoids encompass a diverse range of compounds, including flavanones, which are notably abundant in citrus fruits (such as hesperidin and, to a lesser extent, naringin), and flavanols, which represent the most prevalent quantitative component in numerous vegetables (such as capers, onions, chicory, peas, and blueberries) and are primarily composed of quercetin.

In their natural state, the majority of flavanones are glycosylated, primarily with rutinose and neohesperidose. Among these, hesperidin ((2S)-3′,5-dihydroxy-4′-methoxy-7-[α-L-rhamnopyranosyl-(1 → 6)-β-D-glucopyranosyloxy] flavan-4-one) is the most prevalent polyphenolic compound in citrus fruits. Fresh orange juice typically contains approximately 30 mg of hesperidin per 100 mL [65], although it is present in greater quantities in the white portion of the peel [66]. Consequently, this substance is widely available as a by-product of citrus cultivation and consumption. It can be extracted, purified, and incorporated into dietary supplements or in skin formulations [1].

Hesperidin is defined structurally by an aglycone, known as “hesperetin”, which binds a 6-O-α-L-rhamnosyl-D-glucose moiety at position 7 via a glycosidic bond. Hesperetin is released in the gut by the bacterial flora, which in turn is influenced by the ingestion of hesperidin [67,68].

The radical scavenging property of flavanones is dependent on their structural characteristics. In hesperidin, the hydroxyl groups at the 3′- and 5- positions exhibit mild antioxidant activity. Hesperetin possesses an additional hydroxyl group (7-), which contributes to its enhanced antioxidant activity [16].

Hesperidin has the capacity to penetrate beyond the stratum corneum of the epidermis [69] and exhibits favorable skin permeability [70]. Research was conducted using Bronaugh cells on human dorsal skin. The findings indicated that mangiferin (a natural polyphenol, mainly present in the mango plant) and hesperidin were able to traverse the stratum corneum and penetrate the epidermis and dermis. The aqueous solution of hesperidin exhibited the greatest capacity to permeate the skin, with a concentration of 250.92 +/− 16.01 ng/cm^2^. Moreover, the skin microbiome may also influence the bioavailability of hesperidin when administered orally [71].

Hesperidin has low water solubility and is poorly absorbed in the small intestine, a characteristic shared by many other flavonoids. It is hypothesized that hesperidin has limited bioavailability due to the flavonoid-bound rutinoside moiety. A clinical study in human subjects demonstrated that the removal of the rhamnose group to produce the corresponding flavonoid glycoside (i.e., hesperetin-7-glucoside) enhances the bioavailability of hesperetin aglycone in the small intestine [72]. Therefore, the enhancement of water solubility through methylation or glycosylation optimizes the bioavailability, metabolic stability, and tissue distribution of the compound. Additionally, the methylation of hesperidin results in the formation of HMC, which displays a higher degree of water solubility in comparison to that of hesperidin itself [73,74].

### 5.2. Bibliographic Research

**Figure 8:**
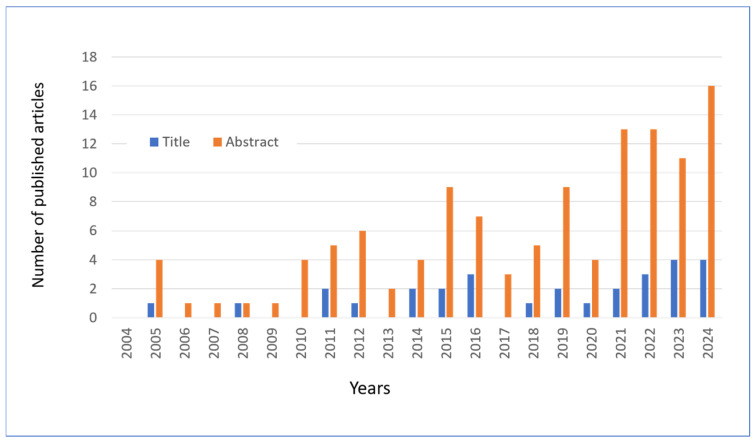
The number of articles published and extracted from PubMed with keywords “Hesperetin” OR “Hesperidin” in the abstract and “Skin” in the title or abstract. Data through to 31 December 2024.

In this review, articles on hesperidin and related molecules published in the last 20 years were searched using the PubMed platform, using the keywords “hesperidin” or “hesperetin” combined with “skin” and the year of publication. Following an initial screening, a total of 135 articles were identified as being of interest to the topic under review. The publication of these articles is distributed over time, as illustrated in Figure 8. In collecting the results, the articles that mentioned the two flavonoids in the title or in the abstract were calculated separately.

In addition to the selection shown in Figure 8, this review also drew on other selections of papers reviewed in the international PubMed database. To illustrate, a search using the primary keywords “Hesperidin or Hesperetin” returned eight articles on “Photoaging since 2016, 22 articles on “Antiaging” since 2006, 17 articles on “UV radiation” since 2000, 18 articles on “Keratinocyte” since 2005, and 57 articles on “Wound healing” since 1997. Additionally, other articles were selected based on citations of original papers or reviews (cross-referencing).

## 6. Experimental Models of Skin Irradiation

The mechanisms by which hesperidin exerts beneficial effects on skin function can be attributed to its antioxidant properties, the inhibition of UV-induced signaling pathways, the stimulation of epidermal proliferation and differentiation, and the modulation of inflammatory processes (see the summary in Figure 6). Given its low cost, wide availability, and superior safety, hesperidin may prove useful in the treatment of a variety of skin conditions.

There are several experimental studies documenting the protective effect of hesperidin against UV-induced damage. These are schematically summarized in Table 1 and briefly described in the following paragraphs, distinguishing the works according to the type of experimental model and following a chronological order where possible.

Hesperidin has been demonstrated to exert protective effects against UVA-induced cell damage in human keratinocytes in vitro [75]. In this study, hesperidin was observed to preserve cell viability and reduce oxidative stress, as evidenced by the levels of superoxide dismutase (SOD), malondialdehyde (MDA) and total antioxidant capacity (T-AOC) (see “A” in Figure 6). Concomitantly, the gene expression of TNF-a, IL-1β, and IL-6 was diminished.

The keratinocytes treated with hesperidin demonstrate enhanced resistance to UVB irradiation in comparison to the untreated cells [86]. The effect was documented as a reduction in DNA damage, lipid peroxidation, and the apoptotic index, and the expression of caspases 3 and 9 (“B” and “C” in Figure 6). In a further experiment, keratinocytes were treated with hesperidin for a period of 24 h, after which they were irradiated with UVA. The treatment demonstrated efficacy in safeguarding cell viability, enhancing SOD levels, curbing MDA production, and attenuating the gene expression of inflammatory cytokines (TNF-α, IL-1β, and IL-6) (“D” in Figure 6). The release of ROS by UVB-irradiated keratinocytes is increased in a dose-dependent manner, while cell proliferation is proportionally reduced, indicating that oxidative stress and cell damage are closely linked [14]. This also demonstrates that oxidative stress is a direct consequence of cell damage, rather than merely of the inflammatory process. The authors demonstrated that a combination of agents, comprising dihydroceramide, niacinamide, resveratrol, glucosyl hesperidin, and phytosterol esters, enhances skin barrier integrity and attenuates the impact of UVB radiation on the skin.

The flavanone glycoside hesperidin has been demonstrated to exert an anti-photoaging effect by reducing the expression of matrix metalloproteinase-9 (*MMP-9*) through MAPK-dependent signaling pathways [84] (“E” in Figure 6). Elderly keratinocytes (HEK001 cells) exposed to UVB light and treated with hesperetin demonstrated increased viability, which correlated with the increased expression of the age-associated genes *CISD2*, *FOXM1*, and *FOXO3a* and decreased oxidative stress (“A” in Figure 6) and *MMPs* (“G” in Figure 6) [40]. Hesperetin is a promising activator of *CISD2*, thereby slowing the ageing process and promoting longevity [39]. The anti-aging effect of hesperetin is primarily dependent on *CISD2*, as the transcriptomic analysis of skeletal muscle indicates that the majority of differentially expressed hesperetin-related genes are regulated by hesperetin in a *CISD2*-dependent manner.

Kim et al., after finding that UVB induces the expression of vascular endothelial growth factor (*VEGF*) and hypoxia-inducible factor (*HIF-1α*) in primary human keratinocytes and fibroblasts, also found that hesperidin reduces UVB-induced *VEGF* expression [76]. The effect is mediated by inhibition of the PI3K/Akt signaling pathway [76] (“F” in Figure 6). These findings are in accordance with those of previous studies [87], which demonstrated that hesperidin hinders the expression of *HIF-1α* in mast cells and the subsequent generation of VEGF. Furthermore, the authors demonstrated that hesperidin inhibits the production of the cytokines IL-1β, IL-8, and TNF-a (“D” in Figure 6). This effect is significant in light of the involvement of mast cells in acute UVB damage, as previously discussed.

The pre-treatment of fibroblasts with hesperetin glucuronide at concentrations of 3 and 30 µM resulted in enhanced resistance to UVA irradiation, as evidenced by a reduction in cell necrosis [77]. The non-glucuronidated form of hesperetin did not exhibit this property. Bae et al. [78] also investigated the protective effect of hesperetin and naringenin from *Citrus unshiu* (satsuma mandarin) extract on fibroblasts exposed to UVA irradiation. Their findings revealed that flavanones increased collagen biosynthesis and decreased the expression of MMPs and β-galactosidase, an enzyme typically associated with cellular senescence [88,89] (“E” in Figure 6).

The objective of a study conducted on rats was to assess the photoprotective efficacy of a topical formulation comprising a gel containing 10% hesperidin [85]. The animals were subjected to UVA-UVB irradiation, and the in vivo results demonstrated that the formulation safeguarded the skin from damage by reducing erythema, lipid peroxidation, and myeloperoxidase activity (a marker of inflammation with leukocyte infiltration) while enhancing the activity of antioxidant enzymes.

### 6.1. The Role of the Nrf2/ARE System

Flavonoids possess a molecular structure that enables them to participate in redox reactions and free radical scavenging, which are involved in the biochemical phenomena described here and in cellular pathology resulting from attack by various pathogens. Hesperidin plays a pivotal role in antioxidant defense systems, working in conjunction with ascorbate and other fat-soluble vitamins (A and E), and has been demonstrated to be an efficacious agent against O_2_^−^ and ˙OH species [90].

The antioxidant action of hesperidin is not limited to a direct “scavenger” mechanism; rather, it is mainly exerted by stimulating endogenous detoxification systems, primarily the Nrf2/ARE system [83,91,92]. Hesperetin has been observed to inhibit the production of NO by microglial cells stimulated by lipopolysaccharide (LPS) [93].

**Figure 9:**
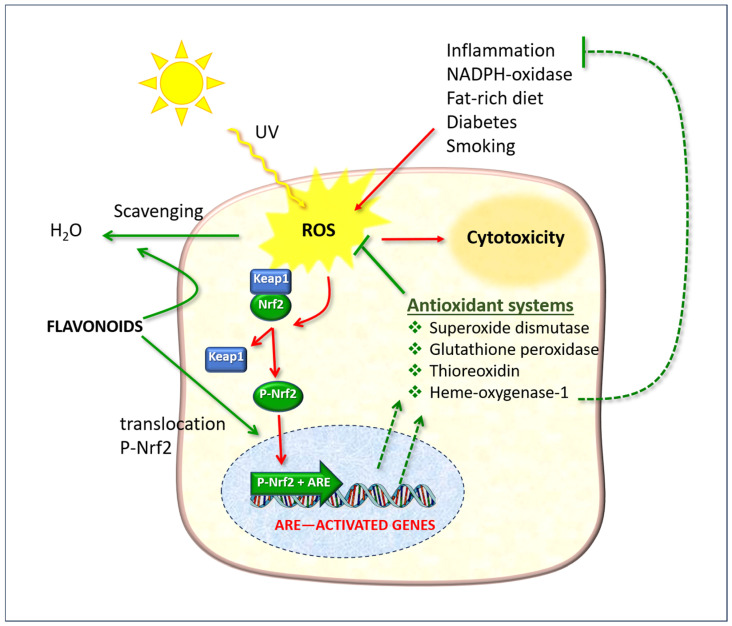
The cellular defense effects of flavonoids, which function as direct free radical scavengers and as stimulants of the Nrf2/ARE pathway. ROS: reactive oxygen species; Keap1: Kelch-like ECH-associated protein 1; Nrf2: nuclear erythroid factor 2-related factor 2. The red arrows indicate phenomena due to oxidative stress; the green arrows indicate protective actions enhanced by flavonoids.

The nuclear factor erythroid 2-related factor 2 (Nrf2) is of particular significance as it regulates gene expression through a promoter sequence known as the antioxidant response element (ARE) (Figure 9). Normally, Nrf2 is bound to another protein called Kelch-like ECH-associated protein 1 (Keap1). In normal physiological conditions, the protein complex is rapidly degraded by the ubiquitination and proteasome system, thereby preventing its functional activity. However, in the presence of ROS, Nrf2 dissociates from Keap1, undergoes phosphorylation, and translocates to the nucleus, where it forms a dimer with a small musculoaponeurotic fibrosarcoma (Maf) protein and binds to ARE upstream of the promoter [94]. This ARE + Nrf2 dimer then initiates the transcription of messenger RNA, resulting in the expression of a number of target genes, including those that encode antioxidant enzymes (illustrated as “Antioxidant systems” in Figure 9).

The capacity of hesperidin to mitigate the effects of toxic ROS damage and promote Nrf2 expression has been documented by several authors in a range of experimental models, particularly in the context of hepatocarcinogenesis [95], hepatotoxicity [96], neuroinflammation, and neurodegeneration [11].

In basal layer keratinocytes, the activation of Nrf2 target genes has been demonstrated to reduce UVB cytotoxicity and enhance ROS detoxification through the activation of cytoprotective genes [97]. Furthermore, the aforementioned authors demonstrated that Nrf2 plays a role in the activation of the production, recycling, and release of glutathione and cysteine by keratinocytes.

Interestingly, Nrf2 also contributes to the expression of the heme oxygenase-1 (HO-1) axis, which in turn regulates the expression of inflammatory mediators, the NF-kappaB pathway, and macrophage metabolism [94]. The NF-ĸB system is a protein complex comprising transcription factors that regulate the expression of genes involved in immunity, inflammation, and oxidative stress responses. From a functional perspective, Nrf2 inhibits oxidative stress-mediated activation of NF-κB by reducing intracellular ROS levels [98] and preventing the nuclear translocation of NF-κB [99]. This provides further evidence of a link between oxidative stress and inflammation and suggests that agents that stimulate Nrf2 indirectly have a modulatory effect on inflammatory processes.

### 6.2. Murine Models

One of the earliest experimental studies was conducted on guinea pigs. The dorsal skin was subjected to UVB radiation for a period of 2 weeks. The topical application of 1% hesperetin for 4 consecutive weeks to the irradiated areas resulted in a reduction in skin irritation, abnormal pigmentation, and transdermal water loss [79].

In a model of UVB-induced DNA damage to the epidermis of Balb/C mice, the formation of cyclobutane pyrimidine dimers (CPDs) in the epidermis was observed by immunohistochemical staining and dot blotting. The topical application of hesperidin was observed to significantly reduce the amount of epidermal CPDs, thereby indicating irradiation-induced damage. The protective effect was accompanied by an increase in p53 protein, which is known to maintain genome stability by preventing mutations [80].

In a study conducted by Petrova et al. [81], mice were treated topically with hesperidin for a period of 10 days, after which they were irradiated with UVB at a dosage of 180 mJ/cm^2^. In comparison to the control group, the administration of hesperidin resulted in a notable reduction in the incidence of skin erythema and oedema, as well as epidermal hyperplasia. From a biochemical perspective, the effect was mediated by a reduction in cellular DNA damage and lipid peroxidation, as well as an increase in catalase and SOD enzyme activities.

One of the consequences of radiation-induced damage to the skin is increased vascularity, which is mainly due to an increased capillary network and the formation of small vessels. Kim et al. [76] investigated the effects of orally administered hesperidin on UVB-induced angiogenesis in hairless mice. The administration of UVB irradiation resulted in an increase in the number and size of cutaneous blood vessels. However, treatment with hesperidin inhibited the phenomenon of neovascularization. The expression of *VEGF* and MMPs (*MMP-13* and *MMP-9*) was induced by UVB radiation; however, these were all inhibited by treatment with hesperidin (“G” in Figure 6). The authors demonstrated that the flavanone exerts its effect also by inhibiting the PI3K/Akt pathway in skin cells in response to UVB radiation (“F” in Figure 6). Additionally, HMC has been shown to possess anti-angiogenic properties in SP-stimulated human skin explants, as evidenced by a reduction in the number of dilated vessels, total vessel area, and chemotactic cytokine IL-8 [100].

The protective effects of oral treatment were investigated by Lee et al. [84]. Mice were irradiated with UVB at increasing doses every 2 days for 12 weeks and administered 100 mg/kg body weight of hesperidin, or water as the control, on a daily basis. The treatment resulted in a reduction in wrinkle formation, trans-epidermal water loss, cytokine expression (“D” in Figure 6), and MMPs expression (“G” in Figure 6). In comparison to the control group, the administration of hesperidin resulted in a reduction in the phosphorylation of MAPK and extracellular signal-regulated kinases, suggesting that flavanone may interfere with this mechanism, which, as previously seen [36], is implicated in radiation damage (“E” in Figure 6).

In transgenic mice, elevated levels of *CISD2* mitigate the age-related dermatological phenotype [40]. In a murine model, specifically using 21-month-old mice, hesperetin (administered interperitoneally at a dose of 10 mg/kg for a period of 7 days) was observed to provide protection against photoaging induced by UVB exposure for a period of 5 days. This protection was accompanied by an increase in *CISD2* expression [40]. The importance of *CISD2* was confirmed in mice with a knockout of this gene, where hesperetin did not improve the skin ageing parameters.

### 6.3. NADPH Oxidases

The enzyme NOX, which is responsible for the production of ROS, was initially identified in the membrane of phagocytic cells [101]. Its primary function is the production of O_2_^−^ and H_2_O_2_ during the process of phagocytosis and the killing of microorganisms. Over time, additional human homologous enzymes have been identified that share the same catalytic subunit of phagocyte NOX, now designated NOX2/gp91(phox) [102,103]. NOX consists of complex multidomain proteins that require different combinations of other proteins for assembly and activity. They are also capable of transporting electrons across the plasma membrane, generating O_2_^−^ and other ROS downstream.

The mechanisms of activation and tissue distribution of different family members are markedly disparate [104]. The physiological functions of NOX family enzymes include host defense, the post-translational processing of proteins, cell signaling, the regulation of gene expression, and cell differentiation. Additionally, NOX enzymes are implicated in a multitude of pathological processes. A deficiency in NOX can result in an increased susceptibility to infection or hypothyroidism. Furthermore, dysregulated increases in NOX activity also contribute to a variety of diseases, particularly cardiovascular disease and neurodegeneration.

The generation of ROS by NOX1 in the skin is stimulated by UVB radiation, suggesting that NOX plays a pivotal role in UV-induced skin carcinogenesis [105]. The irradiation of keratinocytes with UVB radiation has been demonstrated to result in the activation of NOX1, which in turn has been shown to contribute to an increase in apoptosis and carcinogenesis [106]. The authors proposed that the inhibition of NOX1 may represent a promising strategy for the prevention of UV-induced damage to the skin, particularly in patients with deficiencies in DNA repair capacity. Additionally, NOX4, which is induced by bleomycin toxicity, has been identified in the skin [107].

A number of natural polyphenolic compounds have been demonstrated to inhibit NOXs, including berberine, thymoquinone, catechin, quercetin, resveratrol, curcumin, hesperidin, and G-hesperidin [108,109,110,111,112]. The mechanism of action is illustrated schematically in point “H” of Figure 6.

In a mouse model of UVB-induced skin oxidative stress and inflammation [82], the intraperitoneal administration of hesperidin methylchalcone was observed to reduce skin oedema and inflammation. This was evidenced by a reduction in neutrophil migration, lipid peroxidation, leukocyte myeloperoxidase activity, and MMP-9 generation, as well as a decrease in O_2_^−^ generation and the mRNA expression of gp91phox (the major NOX2 subunit). Conversely, there was an increase in glutathione levels and catalase activity.

The same research group [83] tested the antioxidant activity of a topical formulation containing 1% HMC [83]. Local treatment with HMC before and after UVB irradiation resulted in a reduction in skin oedema, lipid peroxidation, O_2_^−^ production, and the inflammatory cytokines IL-6 and TNF-α (“D” in Figure 6). This protective effect was associated with the activation of antioxidant capacity via the mRNA expression of erythroid nuclear factor 2-related factor 2 (Nrf2) and increased HO-1. These findings were replicated on in vitro cell systems.

## 7. Other Anti-Aging and Skin Health Actions

Given the complex nature of skin functions and their dependence on numerous factors, it is evident that UV radiation interacts with environmental pollution and common daily habits (e.g., a poor diet, alcohol consumption, and smoking) in causing damage to the skin. To gain a comprehensive understanding, it is essential to acknowledge the additional benefits that hesperidin and similar molecules offer in terms of skin protection and repair, which complement and reinforce those of UV protection (Table 2).

### 7.1. Enzyme Studies

Hesperidin and hesperetin have been observed to significantly inhibit purified elastase and hyaluronidase enzymes at the lowest concentrations of 5 µM and 0.5 µM, respectively [62]. The modulation of elastase, hyaluronidase, and collagenase activity by the tested substances was evaluated spectrophotometrically using tube assays. Collagenase enzymes are a group of MMPs responsible for collagen degradation and have been linked to an ageing effect on the skin when present at higher levels in the extracellular matrix. The work of Stanisic et al. demonstrates that hesperidin exerts an inhibitory effect on collagenase [16]. Hesperidin reduced enzyme activity, potentially through physical interaction with the protein as evidenced by fluorescence experiments, the chelation of the zinc (II) ion located in the catalytic site of the enzyme, and the alteration of the protein’s conformation [16].

### 7.2. In Vitro Models

In models of skin ageing utilizing physiologically aged human dermal fibroblasts, the application of hesperetin was observed to result in a reduction in the levels of MMP-1 and MMP-2 [62]. The inhibition of neutrophil elastase by hesperidin has also been documented by other researchers [124].

An interesting in vitro experimental model suggests that hesperidin exerts an anti-inflammatory effect on keratinocytes, potentially mediated by its antioxidant properties [120]. Human keratinocytes (HaCaT line) were preincubated with hesperidin (20 µg/mL) prior to treatment with H_2_O_2_, which was used to induce oxidative stress. In comparison to the control cells, the keratinocytes incubated with the hesperidin demonstrated a reduction in the production of IL-8 and TNF-α, as well as a decrease in cyclooxygenase-2 expression. The inhibitory effect was mediated by the blockade of the NF-κB factor, phosphorylated IκBα, and phosphorylated p38 MAPK.

The activation of macrophages, which are ubiquitous cells in connective tissue, including the dermis (Figure 1), is also inhibited by hesperidin or hesperetin (40–100 μM), with a concomitant reduction in the release of prostaglandins, NO, and ROS [121,122]. Moreover, the effects were mediated by the inhibition of NF-κB and IκBα. In a model of psoriatic dermatitis, HaCaT cells were stimulated with LPS and treated with hesperidin (5–20 mg/mL) for 24 h, resulting in the inhibition of the IRS-1/ERK1/2 pathway and proliferation. A mild anti-inflammatory effect of hesperidin and HMC has been demonstrated in numerous experimental systems [7,123,125,126,127,128,129,130].

### 7.3. In Vivo Studies

NC/Nga mice spontaneously develop a form of dermatitis characterized by an overproduction of IgE as they age, which closely resembles atopic dermatitis [131]. Hesperidin and α-glucopyranosyl (αG)-hesperidin were administered to NC/Nga mice in a 0.1% diet for a period of 8 weeks [123]. In addition to the observed reduction in IgE, IL-17, and IFN-γ, it is noteworthy that cytotoxic T lymphocyte antigen 4 (CTLA4), a T cell regulatory marker (Treg), was increased in splenocytes from animals treated with αG-hesperidin feeding. This suggests that this may be the primary mechanism through which the diet-induced rebalancing of the skin immune system occurs.

Hou et al. [113] conducted a study in which they tested the hypothesis that the topical application of hesperidin would improve barrier function in hairless mice, which was measured as transepidermal water loss. Histological analysis revealed evidence of cell proliferation, epidermal differentiation, and the increased secretion of lamellar bodies. Lamellar bodies are elongated structures, measuring approximately 300–400 nm in width and 100–150 nm in length, secreted by keratinocytes in the spinous and granular layers of the epidermis. They form a type of impermeable, lipid-containing membrane that plays a crucial role in maintaining the integrity of the skin barrier.

An increase in lamellar bodies was reported by Man et al. [114] in a mouse model treated topically with corticosteroids (clobetasol propionate), which have been demonstrated to cause the inhibition of epidermal mitosis, differentiation, and lipid production. The topical application of 2% hesperidin, following the administration of steroids for a period of 9 days, also resulted in improvements in barrier function, cell proliferation, and increased glutathione reductase levels. Furthermore, the same research group replicated these findings in aged mice [34], which exhibited thinner and atrophic skin. The topical application of 2% hesperidin twice daily for 9 days was observed to increase lamellar body formation and barrier function, without an accompanying increase in keratinocyte proliferative activity. It is noteworthy that hesperidin has been demonstrated to stimulate the expression of ATP Binding Cassette Subfamily A Member 12 (*ABCA12*), a transmembrane transporter of glycosylceramide, a substance that is essential for lamellar body formation. While the epidermis of aged mice exhibits lower levels of *ABCA12* mRNA than young mice, the topical application of 2% hesperidin for 9 days resulted in a notable elevation in *ABCA12* mRNA expression, reaching the expression levels observed in young mouse skin.

Furthermore, the eye lens is implicated in the process of light-induced ageing, whereby it becomes opaque and may ultimately result in the formation of cataracts. A study conducted by Nakazawa et al. investigated the anti-cataract effects of alpha-glucosyl hesperidin in vitro on human lens epithelial cells and in vivo, utilizing a sodium selenite-induced cataract model in rats [74]. In vitro, the administration of hesperidin (at concentrations of 50 or 100 µM) was observed to reduce the number of cells undergoing apoptosis in lens epithelial cell lines. Rats with induced cataracts were administered alpha-glucosyl hesperidin (200 mg/kg) orally (or saline as a control). The treatment resulted in a notable reduction in cataract severity and a restoration of antioxidant levels to normal. The levels of antioxidants in the lens and plasma decline with age. However, this decline can be reversed by treatment with 1% or 2% glucosyl-hesperidin in a dose and time-dependent manner [132]. In experimental models, glucosyl hesperidin has been demonstrated to be an effective and safe treatment when administered as an eye drop [133] and orally [134].

In a mouse model, encapsulated hesperidin in a hydrogel formulation [135] demonstrated a significantly enhanced wound closure rate in the treated group of 98.96 +/− 1.50% at 14 days post-wounding, as compared to the control group, which exhibited a wound closure rate of 89.12 +/− 2.6%. The synthesis of collagen and cell growth was observed to be higher in the hesperidin formulation than in the untreated animals. Furthermore, the powerful anti-inflammatory and antioxidant effects of HMC have been demonstrated in mouse models of monosodium urate-induced arthritis, in which the oral administration of HMC was observed to stimulate Nrf2/HO-1 mRNA expression and to exhibit a potent anti-inflammatory effect, which is mediated by the inhibition of the NF-kappaB factor [136].

Hesperidin has been demonstrated to positively impact cutaneous circulation [137]. In a double-blind study conducted by the aforementioned authors, 18 healthy adult volunteers were given either a drink containing 100 mg glucosyl hesperidin or a placebo. The test comprised the immersion of the hand in cold water (15 °C) for 5 min, followed by the measurement of blood flow and skin temperature. The administration of glucosyl hesperidin resulted in a notable acceleration in the restoration of skin blood flow and temperature, which had been diminished by the exposure to cold water, in comparison to the effects observed in the placebo group. The same group subsequently published a randomized, double-blind, crossover, placebo-controlled study [138], which confirmed a significant increase in cutaneous blood flow following the ingestion of relatively small doses (20–40 mg) of hesperetin-7-O-glucoside.

The topical application of a serum containing hesperetin 0.1% and cyclic lysophosphatidic acid sodium 0.1% demonstrated favorable effects on stratum corneum hydration and skin elasticity, as determined by objective measurements over a 12-week period [64]. The study, which included a total of 35 female subjects, was not randomized; however, improvements from the baseline were assessed by objective methods. Skin biopsies revealed significant enhancements in hyaluronic acid levels and elastic fiber structure.

Finally, it is interesting to note that hesperetin enacts activity against multiple pox viruses that affect the skin, such as buffalopox virus, vaccinia virus, and lumpy skin disease virus [139]. These authors demonstrated that the treatment of infected Vero cells reduced viral RNA and the interaction between mRNA and the eukaryotic translation initiation factor eIF4E; furthermore, hesperetin reduced buffalopox virus infection in a chicken egg chorioallantoic membrane model.

### 7.4. Hesperidin and Melanogenesis

Melanin is the primary dermal pigment responsible for the pigmentation of the skin and provides protection from UV radiation. Consequently, numerous researchers have investigated the effects of hesperidin on this function, mainly through the utilization of in vitro melanocyte cell lines. The results demonstrated variability depending on the experimental model and dose.

Mouse melanocyte lines incubated with 5–20 μM hesperidin for 3 days [140] or incubated with 20 μg/mL citrus extracts or 3–50 μM hesperetin for 48 h [141,142] demonstrated an increase in their melanin and tyrosinase content, the key enzyme in its production. The same result was observed in human melanocytes [142,143]. Conversely, other findings indicate that hesperetin functions as an inhibitor of tyrosinase [144]. For instance, in a study by Lee et al. [143], the treatment of melanocytes with hesperidin at a concentration of 50 μM for 3 days resulted in a reduction in melanin content and tyrosinase activity. An experiment on a reconstructed human epidermis treated topically with 0.2% hesperidin for 14 days demonstrated a reduction in pigmentation [140]. More recent findings suggest that the enzymatic kinetics of tyrosinase are inhibited by hesperidin and naringenin [145]. The potential application of tyrosinase inhibitors in cosmetics includes the prevention of hyperpigmentation and dyschromia in the skin.

The mechanism by which flavonoids compounds such as the flavanone glycoside hesperidin may contribute to vitiligo treatment is not yet fully understood. However, recent work by Shivasaraun and colleagues [146] suggests that combining flavonoids with trimethylpsoralen in a nanoemulsion-based gel formulation may constitute an effective approach.

## 8. Conclusions

UV radiation has been demonstrated to reduce cell viability, resulting in oxidative stress and a dose- and duration-dependent inflammatory response. A number of studies reviewed here have demonstrated that hesperidin and related molecules may confer beneficial effects on skin health due to their antioxidant, cytoprotective, and anti-inflammatory properties. However, its oral therapeutic use is constrained by its poor water solubility, which consequently results in poor bioavailability. Therefore, topical applications offer the advantage of targeted drug delivery and improve the bioavailability of the active ingredient at the site of action.

In general, hesperidin treatment effectively protects keratinocytes and experimental animals from UV radiation-induced skin damage, with complex molecular mechanisms involving both antioxidant and anti-inflammatory capacities. This experimental basis may therefore stimulate controlled human studies to validate its use not only as a cosmetic but also as a cosmeceutical. The potential for both food and topical applications, coupled with the extensive availability of the raw material as a by-product of citrus processing, would facilitate further investigation and its large-scale utilization.

## Figures and Tables

**Table 1:** Effects of hesperidin and related molecules on experimental models of UV irradiation.

Model	Route	Treatment	Effects	Mechanisms	Ref.
Human keratinocytes (HaCaT) exposed to UVA (10 J/cm^2^)	In vitro	220 µg/mL of pure hesperidin, purity ≥ 98% (commercial product, Procter & Gamble, Cincinnati, OH, USA) applied to the cell culture for 24 h	↑ Cellular vitality ↓ malondialdehyde production	↓ mRNA levels of TNF-α, IL-1β, and IL-6 ↑ SOD activity	[75]
Keratinocytes from an elderly person (HEK001 cells) exposed to UVB (20 J/m^2^)	In vitro	Pre-incubation for 24 h with 1–10 µM pure hesperidin, purity ≥ 98% (Sigma-Aldrich, St. Louis, MO, USA)	Photoprotection	↓ *VEGF* expression ↓ Expression of *MMP-9*, *MMP-13* ↓ PI3K/Akt signaling	[76]
Keratinocytes from an elderly person (HEK001 cells), exposed to UVB (20 J/m^2^)	In vitro	10 µM pure hesperetin, purity ≥ 98% (Sigma-Aldrich) for 24/48 h	↑ Mitochondrial function ↓ Oxidative stress	↑ *CISD2* ↑ *FOXM1* ↑ *FOXO3a* ↓ *MMP-1*	[40]
Human skin fibroblasts (FEK4), exposed to UVA (500 kJ/m^2^)	In vitro	3 or 30 µM hesperetin glucuronide (synthesized and verified by HPLC-DAD, LC-MS/MS) for 18 h	↓ Cellular necrosis	Not determined	[77]
Human dermal fibroblasts irradiated with UVA (10 J/cm^2^)	In vitro	Fermented *Citrus unshiu* peel extract (commercial product; rich in hesperetin and naringenin) for 72 h	Photoprotection ↑ Biosynthesis of collagen	↓ Expression of *MMPs* ↓ Expression of β-galactosidase	[78]
Guinea pig, exposed to UVB for 2 weeks, dose not specified	Topical application	Pure hesperetin 1% in microemulsion (commercial product, supplier not specified)	↓ Skin irritation ↓ Pigmentation ↓ Trans epidermal water loss	Not determined	[79]
Balb/C mouse, exposed once to UVB (180 mJ/cm^2^)	Topical application	1 mg hesperidin in 100 µL acetone/cm^2^ applied once daily for 3 days, purity ≥ 98% (from National Institute for the Control of Pharmaceutical and Biological Products, Beijing, China)	Photoprotection	↑ p53 expression ↓ Cyclobutane-pyrimidine epidermal dimers	[80]
SKH-1 mouse, exposed daily to UVB (180 mJ/cm^2^)	Topical application	30 min pretreatment with hesperidin 3 mg/mL, purity ≥ 98% (commercial product; supplier not specified) for 10 days	↓ Erythema and skin edema ↓ Epidermal hyperplasia ↓ Lipid peroxidation ↓ Inflammation	↑ Catalase and superoxide dismutase activity ↓ DNA damage	[81]
Hairless mouse exposed to UVB (4.14 mJ/cm^2^)	Intraperitoneal injection	300 mg/kg HMC, purity ≥ 98% (Sigma-Aldrich), 1 h before and 7 h after each exposure	↓ Skin edema and neutrophil recruitment ↓ Expression of inflammatory cytokines ↓ Lipid peroxidation	↑ Glutathione levels and catalase activity ↓ Myeloperoxidase ↓ *MMP9* ↓ *NOX*	[82]
SKH-1 hairless mice exposed to UVB (4.14 mJ/cm^2^)	Topical application	1% HMC, purity ≥ 98% (Sigma-Aldrich) in lanette cream base, daily for 5 days	↓ Skin edema ↓ Lipid peroxidation ↑ Antioxidant capacity ↓ O_2_^−^ production	↓ IL-6 and TNF-a ↓ *NOX* ↑ Nrf2 expression ↑ Glutathione peroxidase, glutathione reductase, and heme oxygenase-1	[83]
Hairless male mouse (6-week-old) exposed to UVB (60–90 mJ/cm^2^) 3x/week for 12 weeks	Oral administration	100 mg/kg/day hesperidin, purity ≥ 98% (commercial product, supplier not specified)	↓ Wrinkle formation ↓ Trans epidermal water loss ↓ Cytokine expression ↓ Epidermal hyperplasia	↓ *MMP-9* ↓ Phosphorylation of MAPK and extracellular signal-regulated kinases	[84]
HR-1 hairless mouse exposed to UVB (60–90 mJ/cm^2^), 3×/week for 12 weeks	Oral administration	100 mg/kg/day hesperidin, purity ≥ 98% (commercial product, supplier not specified)	↓ Cutaneous neovascularization	↓ *VEGF* ↓ *MMP-9* and *MMP-13* ↓ Hypoxia-inducible factor 1 (HIF-1) alpha	[76]
Swiss albino mouse exposed to UVB (290–320 nm) for 5 weeks	Topical application	*Citrus sinensis* peel extract (ethanolic commercial extract; 15.53 mg% hesperidin), formulated in lipid nanoparticle cream	Slows down skin photoaging and wrinkles ↑ Antioxidant activity	↑ Collagen and SOD ↓ PGE2, COX2, MMP1, collagenase, and elastase activities.	[17]
C57BL/6 and CISD2-KO mice, exposed to UVB (349 mJ/cm^2^) daily for 5 days during hesperetin treatment	Intraperitoneal injection	2-day pretreatment, then treatment for 5 days during UV exposure with 10 mg/kg/day pure hesperetin, purity ≥ 95% (Sigma-Aldrich) for 7 days	Skin protection from photoaging (thickness)	↑ *CISD2* expression	[40]
Rat exposed to UVA-UVB for 5 consecutive days	Topical application	Pretreatment with 10% hesperetin-based hydrogel, once daily (commercial product, supplier not specified)	Protection of dermal–epidermal tissue ↓ Erythema ↓ Lipid peroxidation	↓ Myeloperoxidase ↑ catalase and superoxide dismutase	[85]

↑: increase of enzyme activity or of gene expression; ↓ decrease of enzyme activity or of gene expression.

**Table 2:** Effects of hesperidin and similar molecules on other experimental models of skin pathology.

Model	Route	Treatment	Effects	Mechanisms	Ref.
ECM enzyme activity	In vitro	Hesperidin and hesperetin purity ≥ 98% (Sigma-Aldrich) dissolved in ethanol (<1% final concentration) and tested in solution at 0.5–100 µM	Inhibition of hyaluronidase and elastase by 60–70%	Not determined	[62]
ECM enzyme activity	In vitro	Commercial cream formulation containing hesperidin (0.08–0.90 mmol/L); tested on human skin samples for enzyme assay. Supplier of hesperidin not specified	Interaction with collagenase and its inhibition	Zinc chelation	[16]
Physiologically aged fibroblasts	In vitro	Hesperidin and hesperetin ≥ 98% purity, commercial grade (Sigma-Aldrich), applied to cells at 0.5–100 µM (final ethanol concentration <1%)	Anti-aging	↓ *MMP-1* and *MMP-2* ↓ Elastase and hyaluronidase	[62]
Skin barrier test in hairless mice	Topical application	2% hesperidin cream, ≥98% purity (cosmetic grade, Procter & Gamble), applied topically twice daily for 6 days	Stimulation of proliferation, epidermal differentiation, and increased secretion of lamellar bodies	↑ Barrier function measured as transepidermal water loss	[113]
Skin barrier test in corticosteroid-treated mice	Topical application	2% hesperidin cream, ≥98% purity (cosmetic grade, Procter & Gamble), applied topically 1 h after 0.05% clobetasol propionate, twice daily for 9 days	↑ Proliferation ↑ Barrier function ↓ pH skin surface	↑ Expression of flaggrin ↑ Formation of lamellar bodies ↑ β-glucocerebrosidase ↑ Glutathione reductase	[114]
Skin barrier test in aged mice	Topical application	2% hesperidin cream, ≥98% purity (Procter & Gamble, cosmetic grade), applied topically twice daily for 9 days	Anti-aging ↑ barrier function ↓ Skin surface pH	↑ Expression of *ABCA12*, *NHE1*, and *PLA2* ↑ Formation of lamellar bodies	[34]
Healing of skin wounds in diabetic rats	Oral administration	Hesperidin (25–100 mg/kg/day); administered once daily for 21 days after skin injury in diabetic rats (source and purity not reported)	↓ Wound closure time Improvement of skin architecture	↑ mRNA expression of *VEGF*, Ang-1/Tie-2, TGF-β, and Smad-2/3 ↑ SOD and GSH ↓ Levels of MDA and NO	[115]
Healing of skin wounds in diabetic rats	Oral administration	Hesperidin (10–80 mg/kg/day) for 20 consecutive days after wound induction in diabetic rats (source and purity not reported)	Healing with wound reduction	↑ *VEGFR1* and *VEGFR2* levels ↓ TNF-α, IL-6 ↑ SOD and GSH ↓ MDA levels	[116]
Healing of skin wounds in mice	Topical application	5% hesperidin or 5% naringin hydrogel (source not reported); applied once daily after wound induction	Reduced the average wound healing time ↓ Lipid peroxidation	↑ Collagen synthesis ↑ GSH and SOD ↓ Expression of NF-kappaB and COX-2	[117]
Healing of skin wounds in gamma-irradiated mice	Oral administration	Hesperidin (100 mg/kg), commercial grade (Sigma-Aldrich); administered orally 1 h prior to irradiation	↑ Contraction of the wound ↓ Wound healing time	↑ NO ↑ DNA synthesis ↑ Collagen ↑ Density of vessels and fibroblasts	[118]
Healing of skin wounds in rabbits	Topical application	Hesperidin 1% (formulation, source, and purity not reported); applied topically on ear wounds in rabbits	Scar was softer and lighter in color	↓ Number of fibroblasts, capillaries, and inflammatory cells	[119]
Human keratinocytes (HaCaT line) exposed to H_2_O_2_ (48 h)	In vitro	Hesperidin 20 µg/mL, ≥98% purity (Sigma-Aldrich), 2 h pre-incubation	↓ IL-8 (protein and mRNA) ↓ TNF-α (protein and mRNA) ↓ COX-2 expression	↓ NF-κB, phosphorylated IκBα, and phosphorylated MAPK p38	[120]
Macrophages RAW 264.7 cell line (mice)	In vitro	Hesperidin (5–250 µg/mL), incubated with LPS-stimulated RAW 264.7 macrophages (commercial source not specified)	↓ NO production induced by lipopolysaccharide	Not determined	[121]
Macrophages RAW 264.7 cell line (mice)	In vitro	Hesperidin or hesperetin (40–100 µM), commercial grade (Sigma-Aldrich); 30 min pre-treatment before LPS stimulation	↓ Oxidative stress ↓ PGE2 ↓ COX-2 expression ↓ NO production	↓ Activation of NF-κB ↓ Phosphorylation of JNK1/2 and p38 ↓ IκBα ↓ mRNA of iNOS	[122]
NC/Nga mouse (spontaneous atopic dermatitis)	Oral administration	Hesperidin or α-glucopyranosyl hesperidin (0.1% in diet for 8 weeks); source and purity not reported	↓ IgE ↓ Dermatitis symptoms	↓ IL-17, IFN-γ ↓ *CTLA4*	[123]
Human skin explants (rosacea pattern)	In vitro	HMC 0.2 mg/mL, pre-treatment of human skin explants followed by Substance P stimulation (24 h). Source and purity not reported	↓ Proportion of dilated vessels ↓ Total surface area of blood vessels	↓ Production of IL-8	[100]

↑: increase of enzyme activity or of gene expression; ↓ decrease of enzyme activity or of gene expression.

## Abbreviations

The following abbreviations are used in this manuscript:

ABCA12	ATP Binding Cassette Subfamily A Member 12
AP-1	Activator Protein 1
ARE	Antioxidant Response Element
CISD2	CDGSH Iron Sulfur Domain-containing Protein 2
CPDs	Cyclobutane Pyrimidine Dimers
CTLA4	Cytotoxic T-Lymphocyte Antigen 4
ECM	Extracellular Matrix
H2O2	Hydrogen Peroxide
HEK001	Elderly keratinocytes
HIF-1α	Hypoxia-Inducible Factor 1-alpha
HMC	Hesperidin Methylchalcone
HO-1	Heme Oxygenase 1
Keap1	Kelch-like ECH-associated Protein 1
LO2H	Lipid Hydroperoxide
LPS	lipopolysaccharide
Maf	Musculoaponeurotic Fibrosarcoma protein (small)
MAPK	Mitogen-Activated Protein Kinase
MCs	Merkel Cells
MCC	Merkel Cell Carcinoma
MDA	Malondialdehyde
MMPs	Matrix Metalloproteinases
NF-κB	Nuclear Factor kappa-light-chain-enhancer of activated B cells
NO	Nitric Oxide
NO3−	Peroxynitrite
NOX	NADPH Oxidase
Nrf2	Nuclear factor erythroid 2-related factor 2
OH	Hydroxyl Radical
O2−	Superoxide anion
PI3K	Phosphoinositide 3-Kinase
PIP2	Phosphatidylinositol 4,5-bisphosphate
PIP3	Phosphatidylinositol 3,4,5-trisphosphate
ROS	Reactive Oxygen Species
SOD	Superoxide Dismutase
T-AOC	Total Antioxidant Capacity
Treg	Regulatory T Cell (T-cell subtype)
UV	Ultraviolet
VEGF	Vascular Endothelial Growth Factor
